# Perspectives of healthcare providers in family planning centers on increasing pre-exposure prophylaxis uptake among women who have migrated from sub-Saharan Africa to France

**DOI:** 10.1371/journal.pone.0325078

**Published:** 2025-06-02

**Authors:** Geoffroy Liegeon, Samantha A. Devlin, Victoria Manda, Julie Castaneda, Myriam Toribio, Sophie Florence, Jessica P. Ridgway, Amy K. Johnson

**Affiliations:** 1 Section of Infectious Diseases and Global Health, Department of Medicine, University of Chicago, Chicago, Illinois, United States of America; 2 Department of Infectious Diseases, Saint-Louis Hospital, Paris, France; 3 Department of Gynecology and Obstetrics, Lariboisière Hospital, Paris, France; 4 Section of Maternal Protection, Family Planning, and Sexual Health Promotion, Department of Seine-Saint-Denis, Seine-Saint-Denis, France; 5 Department of Sexual Health, Public Health Service of Paris, Paris, France; 6 The Potocsnak Family Division of Adolescent and Young Adult Medicine, Ann & Robert H. Lurie Children’s Hospital of Chicago, Chicago, Illinois, United States of America; 7 Feinberg School of Medicine, Northwestern University, Chicago, Illinois, United States of America; University of Zimbabwe Faculty of Medicine: University of Zimbabwe College of Health Sciences, ZIMBABWE

## Abstract

Pre-exposure prophylaxis (PrEP) for HIV remains largely underused among women who have migrated from sub-Saharan Africa (WMSSA), despite their accounting for a significant proportion of new HIV diagnoses in France and Western European countries. To expand PrEP reach, we explored healthcare providers’ perspectives on PrEP implementation within family planning centers (FPCs) in the Paris region through focus groups. The focus group discussion guide and rapid content analysis were informed by the Consolidated Framework for Implementation Research (CFIR) 2.0, which uses five domains to capture implementation determinants (Innovation, Outer Setting, Inner Setting, Individuals, and Implementation Process). Twenty providers participated across five focus groups and one key informant interview (median age 45; 80% women, 70% physicians). Oral PrEP was seen as easy to prescribe, but providers advocated for choices beyond the daily pill for better acceptability. While providers recognized increased HIV prevention needs among WMSSA, they found low PrEP demand among women stemming from a lack of knowledge. Although providers acknowledged that PrEP aligned with FPC missions, they cited significant implementation barriers, including limited resources, staff shortages, insufficient on-site capacity, competing priorities, and physicians being the sole prescribers. Provider-level implementation challenges included insufficient training and discomfort in discussing HIV risk and PrEP with WMSSA. Recommendations for implementing PrEP within FPCs included provider training and mentorship, tailored information campaigns for WMSSA, flexible delivery processes, support groups for women, and authorizing midwives and nurses to prescribe PrEP. These results support the need for tailored and multi-level implementation strategies to increase PrEP uptake among WMSSA attending FPCs in France.

## Introduction

People who have migrated from sub-Saharan Africa (SSA) continue to be disproportionately affected by the HIV epidemic in Western Europe, accounting for 37% of new infections among heterosexual individuals [1]. A significant proportion of these new infections are acquired within European territories during the post-migration period [2,3]. The social and economic challenges faced by sub-Saharan African migrants during their settlement significantly increase their risk of HIV infection, particularly among cisgender women (hereafter referred to as “women”) [4,5]. Despite their increased need for HIV prevention, a complex interplay of individual, social, and structural barriers still limits their access to and use of HIV prevention tools [6,7]. This HIV prevention gap is particularly concerning with regard to the uptake of HIV pre-exposure prophylaxis (PrEP). Although PrEP is an invaluable strategy for meeting the HIV prevention needs of women who have migrated from sub-Saharan Africa (WMSSA), there remains a significant lack of PrEP provision for women in many European countries [7].

In France, the HIV incidence among women has not significantly decreased for over a decade. Women born in SSA still account for nearly 80% of the 1,300 women newly diagnosed with HIV each year [8]. Despite women making up 30% of new HIV infections, they represented only 2.5% of the 42,159 individuals who initiated PrEP in the country from 2016 to 2021 [9]. This substantial inequity in PrEP use relative to their need underscores the urgency for tailored interventions to increase PrEP awareness, access, and uptake. Leveraging existing clinic structures to reach women with HIV prevention needs is a valuable strategy for expanding PrEP uptake.

Family planning centers (FPCs) offer comprehensive care to women, including medical, social, and preventive services, making them ideal settings for integrating PrEP alongside contraception, pregnancy termination, and sexually transmitted infection (STI) services. In France, FPCs operate under an “open-door policy”, serving all women regardless of nationality, income, or social coverage. In the Île-de-France Region (Paris and suburbs), which has the largest migrant population in mainland France, FPCs reach a large population of women born in sub-Saharan Africa. In preliminary work conducted at two FPCs in the Paris region, we used a provider-administered questionnaire to assess HIV prevention needs among WMSSA and estimated that 30–50% of those attending these centers met at least one eligibility criterion for PrEP [10]. Despite the centers’ reach among WMSSA and the potential synergy of integrating PrEP with other sexual and reproductive health (SRH) services, a substantial implementation gap persists, resulting in missed PrEP opportunities among WMSSA.

To address this gap, we designed the *PrEParez-vous!* study, aiming to develop effective implementation strategies to increase PrEP uptake among WMSSA in FPCs of the Paris region. In this article, we present the qualitative results of the focus groups that were conducted with FPC providers as part of formative research to inform the development of appropriate and feasible implementation strategies. As such, these focus groups explored providers’ perspectives on PrEP delivery in their centers and potential strategies for integrating PrEP into family planning services.

## Materials and methods

### Study setting

Potential participants were recruited from the network of 24 Paris and 101 Seine-Saint-Denis public centers offering SRH services for women, two areas in the Île-de-France region with a significant population of African migrants. These SRH services for women are provided by various structures that can be hospital-based or standalone, administered by the city or county, and may cater to other populations in addition to women, particularly in sexual health centers. Some of these centers operate daily, while others are open part-time, such as one or two days per week. Most of them are operated by midwives and nurses, with part-time support from physicians.

### Study design

We conducted a qualitative study using content analysis to understand the knowledge, attitudes, and perspectives toward PrEP for WMSSA among healthcare providers working in FPCs in the Paris region. Eligible participants included those who were 1) a physician, nurse, or midwife at an FPC (either full-time or part-time) in Paris or Seine-Saint-Denis; 2) age 18 years or older; 3) able to speak French; and 4) willing and able to give informed consent. Convenience sampling was used to recruit potential participants. Leaders at the centers sent emails with information about the study to providers via listservs; additionally, the research team (authors GL and VM) approached eligible participants in person and at conferences and meetings and informed them of the study. All participants were screened for eligibility prior to enrollment.

A semi-structured focus group discussion guide was developed based on the Consolidated Framework for Implementation Research (CFIR) 2.0, which has been used in numerous studies regarding providers’ perspectives on PrEP [11–13]. The guide was developed in English (by authors SD, GL, and AJ), reviewed and updated accordingly by the rest of the research team, then translated to French (by author GL). Providers were asked about their awareness and/or knowledge of PrEP, experience (or lack thereof) with prescribing PrEP specifically among WMSSA, facilitators and barriers to offering or discussing PrEP with WMSSA, and recommendations for increasing PrEP awareness and uptake among this key population.

Providers were compensated 50 euros for their participation if they attended the focus group outside of their working hours. This study was approved by the French ethics committee (Comité de Protection des Personnes Sud-Est 3, Date: 10/05/2023, No: 2023-A01817-38) and the Institutional Review Board at Ann & Robert H. Lurie Children’s Hospital of Chicago (Date: 10/27/2023, No: IRB 2024−6494). It also complied with the reference methodology MR-003 of the French National Commission on Informatics and Liberty (CNIL) (Deliberation no. 2018−153).

### Data collection

Eligible participants provided verbal consent and completed a brief demographic survey before focus groups began. All focus groups were facilitated by female healthcare staff, primarily by author VM, with support from author JC. VM is an infectious disease physician, with a specialized interest in increasing PrEP among women in Paris and experience in conducting qualitative research. Prior to the start of the focus groups, the facilitators’ interest in PrEP among women and the objectives of the study were explained to all participants. The primary facilitator (VM) knew three of the participants prior to the focus groups but was unfamiliar with the other participants.

Focus groups were conducted from December 2023 to May 2024. One informant interview was conducted with a participant who was unable to join a focus group. Each focus group lasted 60–90 minutes and was conducted in French and audio recorded. No identifying information such as name or date of birth was collected. The first focus group was conducted in person; all other focus groups occurred virtually. Some members of the research team were present during focus groups (authors GL, SD, and AJ); the facilitator introduced these staff members, explained their role in the research study, and confirmed that their presence was acceptable to participants. All information collected on study participants remained strictly confidential and were pseudonymized to the furthest extent possible.

### Data analysis

Discussions were transcribed by a professional bilingual (English/French) transcriptionist, who transcribed the audio recordings in French and translated them to English. These transcripts were reviewed and verified by the research team. Transcripts were not returned to participants for comment and/or correction. However, participants’ responses were summarized and repeated back to them for clarification during the discussions.

All transcripts were uploaded to Dedoose for analysis [14]. Two coders (SD and AJ) developed a preliminary codebook based on the five domains outlined in the CFIR 2.0 (Innovation; Outer Setting; Inner Setting; Individuals – Characteristics; and Implementation Process), as well as on potential implementation strategies for increasing PrEP uptake among WMSSA. The preliminary codes were reviewed by team members who had experience as infectious disease physicians in the Paris region (VM and GL), who offered context-specific information that led to the development of additional parent and child codes; the codebook was iteratively revised to reflect these changes. The primary coder (SD) then applied the codebook to all 6 transcripts (5 focus group transcripts and 1 informant interview transcript). The secondary coder (AJ) reviewed the code application and noted instances in which there was disagreement. Most divergences occurred due to omission and were rectified to 100% agreement. If consensus could not be reached regarding the application of a specific code(s), the PI (AJ) made the final determination. The primary coder reviewed and updated the code application as needed.

Data saturation was determined when there was enough information collected and no new information emerged that added to an understanding of a category. Major and minor themes were generated based on code application. A COnsolidated criteria for REporting Qualitative research (COREQ) checklist has been included (S1_File). Demographic survey data were analyzed using descriptive statistics.

### Inclusivity in global research

Additional information regarding the ethical, cultural, and scientific considerations specific to inclusivity in global research is included in the Supporting Information (S2_File).

## Results

Our study sample consisted of 20 participants. Nineteen providers participated across five focus groups, and one provider completed a key informant interview. The number of providers per focus group ranged from 2 to 6. Participants’ demographic characteristics are described in Table 1. The median age of participants was 45 years. The median time in their job role was 13 years, and the median time working at their specific center was 5.5 years. The majority of participants were physicians (70%), female (80%), and employed full-time (56%). Three participants (17%) were leaders of their centers.

**Table 1:** Participant demographics.

Characteristics	N = 20
Median Age (IQR)	45 (32-55.5)
Median Years in Job Role* (IQR)	13 (4-27)
Median Years at Center** (IQR)	5.5 (2-10)
Gender – n (%)	
Female	16 (80)
Male	4 (20)
Country of Birth – n (%)	
France	19 (95)
Ukraine	1 (5)
Job Role – n (%)	
Physician	14 (70)
Midwife	4 (20)
Nurse	2 (10)
Job Type**- n (%)	
Part-time	10 (56)
Full-time	8 (44)
Type of Center**ⱡ – n (%)	
Sexual health center	10 (56)
Hospital FPC	4 (22)
City run FPC	4 (22)
Mother and Child protection center (PMI)	3 (17)
County run FPC	2 (11)
Leader/Director of Center** - n (%)	
No	15 (83)
Yes	3 (17)

*Data available for 19 participants

**Data available for 18 participants

ⱡCould select multiple answers.

FPC, family planning center.

We present the major themes according to the CFIR 2.0 domains and corresponding constructs. Full representative quotes can be seen in the Supporting Information (S3_File).

### I. Innovation

The CFIR domain “Innovation” in this study refers to the innovation of PrEP for HIV prevention among WMSSA. Relevant constructs that emerged in the data included complexity and relative advantage.

#### A. Complexity.

Among most providers, PrEP was viewed as “not a problem at the medical level…it’s [not] complicated or scary to prescribe” (FG 3). However, several participants raised concerns that WMSSA would view PrEP as too complex. Providers anticipated that WMSSA would have challenges with daily pill intake and/or disclosure to partners, which may complicate their adherence to PrEP.

#### B. Relative advantage.

Some providers discussed alternative PrEP modalities that could better fit the needs of WMSSA compared to daily oral PrEP. For instance, a few providers expressed hope to “have pills that combine contraceptive and PrEP” (FG 3) or to have PrEP “a bit like for men [gay, bisexual, and other men who have sex with men (GBMSM)] on demand” (FG 2). Additionally, one provider suggested that injectable PrEP (e.g., long-acting cabotegravir) could potentially address the anticipated adherence challenges of daily oral PrEP among WMSSA.

### II. Outer setting

The CFIR domain “Outer Setting” refers to the economic, political, and social contexts within which each FPC operates, including the characteristics of the WMSSA attending these centers. Relevant constructs included WMSSA’s attitudes about PrEP, local conditions that increase HIV vulnerability and further demonstrate the need for increased PrEP uptake among WMSSA, and policies that impact access to and delivery of PrEP within FPCs.

#### A. Local attitudes.

Providers described that WMSSA who receive care at their centers are not aware of PrEP and have not asked for it, stating “there is zero demand because they [WMSSA] don’t know about it” (FG 3). Unlike men, who are perceived to be better informed, providers emphasized WMSSA’s lack of knowledge about PrEP. One provider suggested that WMSSA may be less frequently involved in multiple sexual partnerships, which may result in a lower HIV risk perception. Furthermore, a few providers who had prescribed PrEP to WMSSA indicated that some women had difficulty initiating and adhering to PrEP, as evidenced by their tendency to miss consultations, fail to start the medication, or not renew prescriptions.

#### B. Local conditions.

Despite the observed low demand for PrEP, providers recognized the sociocultural and environmental conditions that impact WMSSA and increase their HIV prevention needs compared to other groups. The most frequently cited factors that contributed to HIV vulnerability among WMSSA, as reported by providers, were engaging in sex work, intimate partner violence (IPV)/forced sex, and partner characteristics. Specifically, providers noted that a woman could have a husband who **“**has several wives back in the home country” (FG 4), further increasing the risk of HIV transmission.

#### C. Policies & laws.

For WMSSA without residence permits, providers highlighted the significant impact of the asylum process on their vulnerability. While awaiting asylum, women can access basic needs such as shelter, health insurance, and state aid. However, if their asylum application is rejected, they lose all these rights, leaving them in a highly vulnerable position and increasing their risk of exploitation and HIV infection. Providers noted that to increase the number of PrEP prescriptions among WMSSA, they would “need some more funds and have an outpatient pass [free medical services for WMSSA who have not yet received basic insurance coverage]” (FG 3).

In terms of policies impacting providers, participants highlighted that restricting PrEP prescription to physicians alone poses challenges for some FPCs, which are run by midwives and nurses with only part-time physician support. Providers emphasized if “midwives can initiate PrEP, [that] would be a plus” (FG 1) and expressed support for developing interprofessional cooperation protocols enabling nurses and midwives to prescribe PrEP.

### III. Inner setting

The CFIR domain “Inner Setting” refers to the structural, political, and cultural contexts within which the innovation is implemented, in this case, within the FPCs. Relevant constructs included available resources, structural characteristics, relational connections, and mission alignment/relative priority.

#### A. Available resources.

Providers emphasized the importance of adequate infrastructure and streamlined processes for the effective delivery of PrEP. The availability of resources needed for PrEP implementation among WMSSA varied according to the type of center at which participants were employed. While some centers have “everything on site”, other providers said they “don’t have a lab, we can’t do any blood tests on site, so already, that can be a little obstacle (FG 5).” This absence poses substantial challenges to PrEP implementation, as these centers must refer patients to external facilities for necessary tests. The majority of providers also reported lacking specific resources, such as PrEP informational materials tailored to women.

#### B. Structural characteristics – Work infrastructure.

Most providers acknowledged a significant gap in the integration of PrEP into routine clinical practice within their center. This is attributed to several factors, including limited time and resources for PrEP-related training and education, limited staffing, such as not having “nurses who take samples” (FG 5), and the lack of established prescribing practices or a standardized protocol for assessing HIV risk or delivering PrEP. These problems usually led participants to refer WMSSA to other centers rather than initiate PrEP at their facility.

#### C. Relational connections.

Providers were willing to “jump into the deep end” (FG 5) and start prescribing PrEP, but they emphasized the importance of support from experienced colleagues to guide and reassure them, especially during their first initiations. Having trained colleagues was identified as a factor that created a supportive environment, likely enhancing providers’ confidence and competence in prescribing PrEP. Additionally, participants noted that having multiple providers within one center who are capable of discussing and prescribing PrEP would be beneficial for WMSSA as well because “if one of the staff isn’t [t]here, another is trained and will provide the right care” (FG 2).

#### D. Mission alignment/relative priority.

Participants recognized the urgent need for improving PrEP uptake among WMSSA due to the high prevalence of HIV and increased susceptibility to HIV within this group. However, some providers indicated that increasing PrEP among WMSSA is not considered a priority at their particular center, primarily due to a lack of resources that would be required to address this need and other competing services (i.e., abortions, contraception). Providers reported “it’s not that we don’t want to do it [integrate and prescribe PrEP within their center], but it’s not really in our usual practices” (FG 3).

### IV. Individuals – Characteristics

The CFIR domain “Individuals” and subdomain “Characteristics” refer to the roles and characteristics of the providers and other staff at the FPCs who would take part in the implementation process. The relevant construct included the capability of providers regarding PrEP implementation among WMSSA.

#### A. Capability.

Although there was consensus regarding high comfort levels in discussing PrEP with GBMSM, the majority of providers reported low confidence in their ability to discuss HIV risk and PrEP with WMSSA, citing insufficient cultural sensitivity or competency. One provider stated, “I’m comfortable talking about PrEP because I prescribe it for men. But…I don’t have the reflex to talk to women about it, whereas with men I’m proactive, and that does trigger prescriptions. For women, I’m not proactive and there’s no subsequent prescription” (FG 4). Providers highlighted difficulties bringing up PrEP with women in long-term relationships where infidelity or partner risk may be suspected, due to a reluctance to broach sensitive subjects and concerns about appearing judgmental.

Providers also underscored the stark contrast in messaging between promoting a healthy, fulfilling sex life for GBMSM and mitigating the risks associated with sexual violence and exploitation in WMSSA. While PrEP is seen as a tool for sexual empowerment among GBMSM, healthcare providers view it more as a “damage control” (FG 3) strategy for WMSSA, aiming to reduce HIV infection risks associated with their post-migration vulnerabilities. The specific risk of sexual violence and exploitation among WMSSA creates a substantial barrier for providers in effectively discussing PrEP, as it necessitates addressing a sensitive issue that can foster a negative mindset around PrEP use in women. A participant emphasized the importance of gaining competence in talking about sexual health topics and PrEP with WMSSA because “when you have a medical professional who is very comfortable with PrEP, the lady won’t be against [it]” (FG 5).

### V. Implementation process

The CFIR domain “Implementation Process” refers to the activities and strategies likely to increase PrEP uptake among WMSSA in FPCs. Relevant constructs included assessing the context of the FPCs and tailoring PrEP implementation strategies for WMSSA accordingly.

#### A. Assessing context.

Barriers to PrEP implementation among WMSSA included time constraints for patient education, staff shortages (e.g., social workers, nurses, etc.), limited supplies of PrEP medication at their center, and complex paperwork for women without insurance coverage. The process of initiating PrEP was described as “a bit complicated, it’s a lot of paperwork, things to explain to people. The ladies are lost and, frankly, we’re a bit lost too. It’s not easy” (FG 3). Providers also stated that “it’s not possible to do everything in one go [talk about PrEP and start a woman on PrEP in one visit]…there’s a lot of things to cover. It’s true that it’s too much information for the women” (FG 4).

Facilitators to PrEP implementation among WMSSA included task shifting (i.e., midwives and nurses having the ability to initiate PrEP prescriptions), coordination/support from additional staff members (e.g., social workers, family planning counselors), and flexibility/diversity in the setting of PrEP delivery to accommodate patients’ preferences. One participant stated, “so much the better if these colleagues [midwives and nurses] can actually participate in writing prescriptions and doing the follow-up for the ‘PrEPers’ [people who are using PrEP]” (FG 5).

#### B. Tailoring strategies.

Providers discussed the importance of disseminating information specifically tailored to WMSSA through public campaigns (i.e., bus advertisements, TV commercials, etc.) and posters and videos at their centers. However, one participant specifically noted the need to “be careful in how we formulate [advertising campaigns] because these women are already quite stigmatized, but a communication campaign could be a good thing” (FG 1). In addition to these efforts, participants also emphasized the usefulness of organizing round tables/support groups for WMSSA led by non-physicians because “midwives are more aware of the stories and customs of migrant women” and “maybe the women would be a little more comfortable with [midwives and family planning counselors] and feel free to speak up” (FG 4).

## Discussion

Family planning centers have a crucial role in reaching women to promote and initiate PrEP, particularly among those born in SSA who bear a disproportionate burden of new HIV infections in Western Europe. Health providers within these centers are vital to service delivery, so understanding their insights on barriers and enabling factors for providing quality, integrated PrEP services is essential. Providers in FPCs within the Paris region expressed some ambivalence about integrating PrEP into SRH services. While they acknowledged the urgent need to offer PrEP and saw its potential benefits for WMSSA, they also had concerns about the anticipated acceptability of oral PrEP among women and the additional capacity, time, and resources required for integration into existing services.

The low demand for PrEP among WMSSA attending these centers contributed to the feeling among professionals that PrEP may not be suitable or a priority for these women. At the provider level, the complexity of implementing PrEP was perceived as primarily linked to insufficient training and communication challenges with WMSSA. Structural issues related to the physician’s role as the sole prescriber, variations in resources between centers, centers’ competing priorities, and overall staff shortages also emerged as significant barriers to implementation. As a result, while PrEP was recognized as aligning with the FPCs’ mission, providers’ ability to offer PrEP could be limited unless targeted interventions supporting its integration are implemented.

Our findings align with other studies on HIV and SRH services integration in Africa and the United States, which found that health providers generally supported integration as beneficial but also expressed major concerns about the potential for increased workload and the lack of resources and dedicated support [15–19]. Additionally, the participants in our study indicated that cultural differences with women born in SSA posed an additional barrier for providers, who expressed concerns about “offending her sensitivity” when discussing sexual health and HIV risk and prevention. The low level of comfort and limited cultural sensitivity or competency in discussing HIV risk and PrEP have been documented in other settings, notably among Black/African American women in the U.S. [12,13]. Furthermore, the necessity of addressing the specific risks of sexual violence and exploitation faced by migrant women could also deter some providers in the absence of specific training. These factors may explain why professionals struggle to offer PrEP to women, despite our data indicating that 30–50% of WMSSA attending these centers are potentially eligible [10]. These missed opportunities to initiate PrEP are also influenced by gender bias, with providers less frequently perceiving women as being at risk of HIV infection. The comparison with GBMSM was particularly illuminating in focus groups, as providers reported being much more comfortable discussing PrEP with GBMSM than with women, a finding consistent with previous studies [12]. These results placed the women-provider relationship at the center of the implementation challenge.

Our results are particularly enlightening when contrasted with women’s perceptions of PrEP. While providers anticipated low acceptability, our data from focus groups and surveys among WMSSA attending FPCs revealed enthusiasm for this prevention tool, with a significant proportion of women expressing willingness to try it despite the constraints of daily pill intake [10,20]. Our qualitative analysis among WMSSA also found a high level of satisfaction with the care received at FPCs and a strong trust in FPC providers, regardless of their provider’s cultural background [10,20]. Consistent with other studies, women also emphasized the importance of support and tailored guidance from their FPC providers [13,21]. They viewed FPC providers as their preferred source of PrEP information and identified FPCs as the most suitable place for delivering PrEP to women [10,20]. Thus, it appears that the barriers professionals perceive regarding women’s acceptability of PrEP and cultural sensitivity to HIV risk and PrEP does not fully align with the experiences of women attending these centers. This underlines the need to place the women-provider relationship at the center of the implementation process by addressing and deconstructing specific barriers that exist on the professional side.

While the study revealed numerous barriers, providers also proposed potential strategies to facilitate the integration of PrEP into FPCs and leverage their experience. First, providers underscored the critical need to more widely inform WMSSA about PrEP, using tailored media such as brochures, posters, or videos, both within and outside their centers, to generate demand for PrEP among women. This approach is likely to facilitate PrEP discussions, encourage providers to issue prescriptions, and ultimately accelerate PrEP delivery in FPCs. Second, participants identified a significant knowledge gap concerning PrEP in women and called for dedicated training among providers. Our findings underscore the importance of enhancing communication skills and cultural competence as a fundamental aspect of this training, which should be extended beyond prescribers, given the crucial role of midwives and non-medical professionals in advocating for PrEP in these centers. However, training alone may be not sufficient to enable providers to routinely and independently deliver PrEP [22], and providers could also benefit from additional decision-making support tools, such as a PrEP rapid assessment tool or optimization of the electronic health record, to assist in identifying women who need PrEP and to facilitate discussions with them [23,24]. Lastly, providers’ declarations highlighted the need of having a dedicated healthcare professional who can serve as a referent and leader for PrEP within the center. A “clinical champion” can provide experienced support and guidance, boost provider confidence and competence, and drive successful PrEP implementation. Previous studies have shown the critical role of clinical champions in implementing PrEP in various settings [25–27]. To ensure the responsibility for prescribing PrEP is not perceived as external, the clinical champion should be an internal member of the center and have dedicated and protected time to fulfill this role effectively [22].

Providers also identified other key implementation factors that varied significantly between centers. While staff shortages seemed to be a fundamental problem for all centers, there were significant disparities among centers in terms of workflow, laboratory testing capacity, on-site pharmacy availability, and providers’ prior experience with PrEP delivery. Consequently, centers vary in their readiness to implement PrEP. Sexual health centers that have recently integrated SRH services for women are closest to being able to deliver PrEP to women, given their experience in providing PrEP to GBMSM. These disparities in experience and resources among centers can significantly influence the implementation process. Acknowledging and addressing these disparities is critical for developing a nuanced and adaptable implementation strategy that meets the unique needs of each center.

Finally, our findings emphasize the unrealistic expectation that FPCs can effectively deliver PrEP to women without additional resources. Evidence from other implementation projects indicates that high provider workloads can hinder successful implementation. If providers and centers are already operating at full capacity, PrEP integration is unlikely to be achieved. If hiring additional staff is not feasible, other interventions might be used to facilitate PrEP delivery. Strategies such as task-shifting, where certain responsibilities are delegated from physicians to other healthcare professionals or the implementation of client-centered interventions like HIV self-testing or mobile health approaches to reduce consultation time, have proven effective in streamlining provider workflow in both high- and limited-resource settings [28–31]. In the specific French context, the possibility of expanding PrEP prescription capacity to other healthcare professionals (i.e., midwives and nurses) through interprofessional cooperation protocols was identified across focus groups as a crucial measure to enhance PrEP delivery in FPCs.

Our study has a few limitations to acknowledge. First, our findings are likely biased toward providers who were interested in implementing or improving PrEP delivery within their centers. Therefore, the perceptions of this limited sample may not reflect the views of all providers in FPCs across the Paris region. Moreover, our sample was mostly composed of physicians compared to midwives or nurses. However, it was crucial to prominently represent the insights of physicians, as they are the only providers in France who are currently authorized to prescribe PrEP, and they typically hold leadership roles within FPCs that make them the primary catalysts for implementing changes within these centers. Second, our findings are specific to the geographical context of the Île-de-France region, an area with a high migrant population density where centers serve a significant proportion of WMSSA. Therefore, our findings may not be transferable to other FPCs in France with different organizations, sizes, and patient populations. Lastly, various inherent biases associated with qualitative research, including those introduced by facilitators during focus groups or by coders during the analysis process, as well as social desirability bias, may have influenced the study findings. To enhance the rigor of our study, we used the COREQ checklist for reporting.

## Conclusion

Providers working in FPCs in the Paris region recognized the importance of integrating PrEP with SRH services but expressed concerns about oral PrEP acceptability among WMSSA and the additional resources required for implementation. Provider-level implementation challenges included insufficient training and communication difficulties with WMSSA, with the low demand for PrEP among women as a contributing factor. Structural issues related to variations in centers’ resources, staff shortages, on-site capacity, and having physicians as the sole prescribers also emerged as significant barriers to implementation. To effectively implement PrEP within FPCs, participants recommended provider training and mentorship, tailored information campaigns and support groups for WMSSA, flexible delivery processes, and authorization for midwives and nurses to prescribe PrEP. These results support the need for tailored and multi-level implementation strategies to increase PrEP uptake among WMSSA attending FPCs in France.

## Supporting information

S1 File COREQ (COnsolidated criteria for REporting Qualitative research) Checklist. Completed checklist for reporting qualitative research.
(PDF)

S2 File Inclusivity in Global Research Checklist. Completed checklist for ethical, cultural, and scientific considerations specific to inclusivity in global research.
(DOCX)

S3 File Quotations Table. Representative quotations organized by CFIR domain and corresponding construct.
(DOCX)
