# Supplementary material for: Perspectives of healthcare providers in family planning centers on increasing pre-exposure prophylaxis uptake among women who have migrated from sub-Saharan Africa to France
Source: PLoS One. 2025 Jun 2;20(6):e0325078. doi: 10.1371/journal.pone.0325078 (PMC12129141; doi:10.1371/journal.pone.0325078)
Supplement: S3 File — Representative quotations organized by CFIR domain and corresponding construct. (DOCX) [file pone.0325078.s003.docx]

**Representative quotations organized by CFIR domain and corresponding construct**

| **CFIR domain and construct(s)** | **Themes** | **Representative quotation(s)** |
| --- | --- | --- |
| I. Innovation | | |
| a. Complexity | PrEP is medically simple | “In fact, for me medically it's [PrEP] not complicated, you get it right, it's not complicated. It's like everything, you have to get on with it, but it's rather an ethical, cultural, and human issue.” (FG 3) |
| b. Relative advantage | Injectable PrEP or a dual prevention pill of birth control and PrEP could better meet the needs of WMSSA compared to daily oral PrEP | “The women have enough on their plate because they always worry about having to take a pill every day, they wonder how they can manage that. It’s a constraint just like the contraceptive pill...So now, with PrEP since it’s a pill they must take every day, they see it as a chore. And what’s more, they can’t tell their husbands, so how can they manage it?” (FG 2)  “When we talk about sexuality, we identify two possible risks or consequences: STIs [sexually transmitted infections] and pregnancy. If we could combine the two on a daily dose, then I think it would make sense to offer PrEP at the family planning center since the women initially come for contraception. In that way, we can propose and prescribe PrEP from the outset. Two in one, that would be even more effective.” (FG 3)  “What’s the situation concerning injectable therapy…injectable PrEP, couldn't it be an interesting tool for this kind of demographic? To catch them once every two months to give them their injections?” (FG 1) |
| II. Outer Setting | | |
| a. Local attitudes | WMSSA demonstrate minimal PrEP interest, knowledge, and/or demand | “So, I find that it's not necessarily women who ask for PrEP, because for me, PrEP is more linked to multiple partner activity and there is not necessarily this demand there either.” (FG 3)  “They [WMSSA] don’t know about PrEP, unlike the men who do” (FG 2).  “Finally, it’s my personal experience that there’s no good compliance with the treatment. On the other hand, I see my colleagues who prescribe PrEP for men, and it works well.” (FG 5) |
| b. Local conditions | Experiences of WMSSA that increase their vulnerability to HIV and underscore the importance of implementing PrEP within FPCs | “Of the three-quarters of the women we receive, they have a migratory background, and as a result, we know that three-quarters have suffered sexual violence or that they have had multiple partners often to get a roof over their head. So yes, for me, they are more at risk and often we have a few where we feel that they are indeed accommodated in exchange for sexual relations and use no protection. And then in [the] 18th arrondissement, we have sex workers, Nigerian women, who are more at risk.” (FG 2) |
| c. Policies & laws | Impact of migration policies on women’s vulnerability and HIV risk; impact of policies limiting PrEP prescription to physicians only | “It’s women on the street [who come to the center for care]. There are shelters for women who have applied for asylum and while waiting for the paperwork they are housed, have social security, have a small amount of money. But these women are not granted asylum, there is no political asylum for them, it’s a domestic asylum…When they leave the shelters, there are what I call predators…the men either put them to work on the sidewalk, or they rape them, they kidnap them, and that's when they get AIDS.” (FG 3)  “It's good for our midwives, they’ll have to understand that this is part of their future mission in our centers now, it must be laid out in the interprofessional cooperation protocols.” (FG 5)  *A recent* *interprofessional cooperation protocol in Paris public hospitals authorizes midwives and nurses to initiate PrEP under physician supervision after completing 60 hours of theoretical coursework and practical training. This training is only available to staff working in FPCs affiliated with the Paris public hospitals network (AP-HP).* |
| III. Inner Setting | | |
| a. Available resources | Availability of resources is dependent on the type of center where providers work | “At [a major sexual health center in Paris], it runs like clockwork…On the other hand, [at the FPC], there are problems of means, availability, medication, and all that. So, when we prescribe PrEP, for the moment, we see it more at the [redacted] because they have all the blood samples and now we’ve changed laboratories, so it’s no longer possible to do everything here [at the FPC]…we only have two boxes [of PrEP], which we have to bring in ourselves, that’s it..” (FG 4)  “We have posters, but we don't have documentation aimed at women, it’s just about PrEP in general and women don't feel targeted or concerned.” (FG 4) |
| b. Structural characteristics – Work infrastructure | Most centers do not have the infrastructure (i.e., staff and protocols) required for integrating PrEP into their standardized practice | “If we talk about it and the patients request PrEP, we refer them to these consultations, but there’s no fixed protocol [for assessing a woman’s risk for HIV].” (FG 1) |
| c. Relational connections | Importance of having the ability to consult colleagues/partners for PrEP support | “I've never had spontaneous requests from patients but honestly if it happened, I'd go for it. However, I’d call a partner at the sexual health center to reassure me. I think it's important when you're starting out, to also know that you have partners on whom you can call to get live information if needed. From now on, I have the choice of either calling a colleague at the CEGID [sexual health center] to double check, or simply start the prescription knowing that I’ve already been trained a little bit.” (FG 3) |
| d. Mission alignment/Relative priority | Recognition of need for PrEP among WMSSA but low priority at some centers due to lack of resources and/or competing interests | “We identify a woman who would benefit from PrEP and as we don’t have a lot of time for PrEP consultations in our organization, we’d send her to [a sexual health center], which is a pretty good place for initiating PrEP as it’s their daily ‘bread and butter’” (FG 5).  “And in any case, it’s seriously being considered [staring patients on PrEP]. There’s been a reflection on how to set up consultation times, but unfortunately, it’s to the detriment of general medicine. And that’s a bit of a problem.” (FG 2)  “It depends on the practice. I do abortions in my practice, and it [PrEP] might not be the right fit for the people who are being followed. At least at the family planning center, even for me it makes no sense not to do it and orient ourselves to a sexual health center or elsewhere” (FG 1). |
| IV. Individuals – Characteristics | | |
| a. Capability | Most providers struggle to discuss PrEP with WMSSA and have limited PrEP knowledge in regard to women | “I never have the nerve to talk about it [PrEP] with these women. But regarding the cultural aspect, I’m still dealing with this question. I’m a white doctor, in front of a Black woman and that’s always a thing. I don’t know what kind of experience she’s had with white doctors since she arrived in France” (FG 1).  “I find it difficult to propose PrEP and I know that I should, but I don't know how to bring it up. Sometimes we have patients who have been in a relationship for a long time, who tell me that they've been together for ten years, but hey, the husband lives a little there, he lives a little elsewhere and so I don't know how to raise the subject. Does he have other women? I don’t want to judge people. And as a result, these are women who fall through the testing system because we think, well in fact they've been in a relationship for a long time, so we don't offer PrEP to them. But while we know very well the risks, when they tell us that their husband doesn't live with them, is he cheating on her on the side? You see. And there are some women who know their husbands see other women. And these patients, slip through the cracks, and it's a shame because they're the ones who are potentially the prime candidates for PrEP. Also, when I worked in the hospital, there were patients who sometimes at the beginning of pregnancy were HIV-negative and then they became HIV-positive even though they had the same partner.” (FG 2)  “But in truth, we as professionals, we hardly know about it [PrEP]. In fact, we target it for men with multiple partners. I don't know anyone who has prescribed it for women, at least not in our circle. We're going to have to change our attitudes.” (FG 3) |
| V. Implementation Process | | |
| a. Assessing context (barriers and facilitators) | Barriers included PrEP unavailability on site, staff shortages, and time constraints | “The implementation of PrEP takes a lot of time, you have to explain, you have to reassure, so you have to plan time, but then we hope the ladies will come back.” (FG 5)  “There are not enough of us [staff] for this kind of follow-up [PrEP care]…they’re already struggling to fill [other] interventions” (FG 2).  “For women who are not covered by social security, we keep a minimum of medication but we don't have a lot of it. We have prescription pads to write free prescriptions for STIs, and then the girl goes to the chemist…to get the medicines. We often work like that, we have a little bit of medicine, but not much.” (FG 4)  “We don't have on-site blood tests, and it's true that as soon as we tell people, well, we're prescribing PrEP, you have to go to the pharmacy to get it, then go to the laboratory, do the bio, etc. All this increases the risks of non-adhesion to the treatment. So being able to do everything in one place would be a plus.” (FG 3) |
|  | Facilitators included interprofessional cooperation protocol for non-physicians to prescribe PrEP and coordination/support from additional staff members, and flexibility/diversity in the setting of PrEP delivery to accommodate patients’ preferences | “We have a nursing cooperation protocol [interprofessional cooperation protocol], so nurses prescribe and renew PrEP under medical delegation.” (FG 2)  “During her [midwife’s] gynecological or abortion consultations, she could introduce the notion of PrEP, since she already deals with contraception, depending on what the patients say to her. The midwife is highly trained as she participates in various training courses on violence against women, etc. So, she's really very well informed about it, she's very vigilant, and she's thoughtful about women’s circumstances.” (Key Informant Interview)  “We could very well imagine that, if we want to, us, the practitioners and midwives at the center in PrEP, could set up a protocol and that's it, off we go. The premises, the medical and paramedical staff are all in place.” (FG 5)  “Knowing that there are people who can’t always get to the hospital because they can’t get around in terms of transport, the family units are a good option…I think we can put PrEP into these three areas: gynecology, general medicine, and PrEP. I think it’s possible…even us as nurses could perhaps possibly do a first telephone call with standard prescriptions ready, so that the patients could do their blood test beforehand, possibly get the results and then give them an appointment to start the process…that it, if we’re also given the means, of course, if there is support…and the will is important too.” (Key Informant Interview)  “Whether it’s in a CEGID [sexual health center] or in another place, I think the main thing is that the patient is comfortable with the place she goes to and that she knows the system. In fact, the most important thing is that she has access to exactly where she wants to go, if she’s used to going to a CEGID, we can provide that and if she wants to go to another place, to another hospital, we can provide that, otherwise it holds back the patients. So, really the flexibility of access for the patient is important.” |
| b. Tailoring strategies | Increased dissemination of PrEP information that is specifically tailored to WMSSA; round tables/support groups led by midwives or family planning counselors | “PrEP is very stereotypically associated with MSM [men who have sex with men] and sex workers, and I think that even for heterosexuals, they don’t clearly identify the risks. We need to widen the discussion about PrEP, it's for everyone and not necessarily just for MSM. I think right now it's very much identified with MSM and even a straight man doesn't ask too much about PrEP.” (FG 1)  “As for [family planning] counselors or a nurse, ok but I think we have to work on the communication aspect. It worked well for men; posters in the subway, etc. I think it was very much in demand by the male homosexual communities and we saw patients turn up because they were informed by social networks, through a non-medical but broader communication strategy. We talked about it, we didn’t make a big drama out of it, we showed that it was accessible, and the message got through. So, we could do the same for women.” (FG 4)  “I think there should be a lot more dissemination of information. I believe that if people come with questions and ask our opinion, as professionals we will always be willing to go ahead and prescribe, which is not the same thing as when we have to start the conversation and talk, talk, talk about it until women get on board. I think we're going to get discouraged by talking into the wind, whereas if the information came from other sources than us such [as] posters in the center or a cultural mediator that would be better…we need information on a larger scale…whether the public is informed in one way or another, information campaigns could be on public transport…let people know about PrEP before they even come to us.” (FG 3) |
